# Diaphragmatic Nerve Paralysis After Redo Aortic Valve Replacement That Improved Over Time and Led to Successful Ventilator Weaning: A Case Report

**DOI:** 10.7759/cureus.74783

**Published:** 2024-11-29

**Authors:** Tomohiro Nakajima, Yutaka Iba, Tsuyoshi Shibata, Takeo Hasegawa, Nobuyoshi Kawaharada

**Affiliations:** 1 Cardiovascular Surgery, Sapporo Medical University, Sapporo, JPN; 2 Clinical Engineering, Sapporo Medical University, Sapporo, JPN

**Keywords:** aortic valve replacement, diaphragmatic nerve paralysis, redo, rehabilitation, ventilation

## Abstract

We report a 75-year-old female with a history of two heart operations: aortic valve replacement (St. Jude Medical^TM^ 21 mm) at the age of 44 years for severe rheumatic aortic stenosis and mitral valve replacement (Carbomedics^TM^ 29 mm) at the age of 51 years for rheumatic mitral regurgitation. Decades later, she presented with exertional dyspnea. Echocardiography revealed aortic stenosis with an effective orifice area of 0.79 cm². Coronary angiography showed #6 75% stenosis and a limited mechanical valve opening. After a thorough discussion, the patient agreed to undergo redo surgery.

The surgery involved re-median sternotomy, left internal thoracic artery (LITA) harvesting, pannus removal, and replacement of the aortic valve with a 20 mm ATS advanced performance (AP) prosthesis (ATS Medical, Minneapolis, MN) in a supra-annular position. The LITA-left anterior descending (LAD) bypass was completed, and the patient was weaned from the cardiopulmonary bypass without complications. Postoperatively, the right phrenic nerve paralysis caused transient respiratory challenges requiring tracheotomy and prolonged ventilation. Rehabilitation improved diaphragmatic function and respiratory independence. At six months, the right phrenic nerve function had recovered, and the patient resumed walking independently with a cane. Two years postoperatively, the patient remained ambulatory and attended independent outpatient follow-ups. This report highlights the potential for gradual recovery from phrenic nerve paralysis following open heart surgery, emphasizing the importance of long-term multidisciplinary care.

## Introduction

Phrenic nerve palsy, although uncommon, is a significant complication associated with cardiac surgery, especially during redo procedures. The close anatomical relationship between the phrenic nerve and the pericardium renders it vulnerable to injury during adhesiolysis or dissection. While direct injury or thermal damage during surgery is well-recognized, transient palsy can also occur due to intraoperative edema, ischemia, or inflammation [1]. Phrenic nerve injury may result in diaphragmatic paralysis, which can lead to postoperative respiratory failure, prolonged ventilation, and impaired recovery [2].

Redo cardiac surgery presents additional risks due to the presence of dense adhesions, altered anatomical landmarks, and increased surgical complexity. These factors not only challenge the technical aspects of surgery but also elevate the likelihood of inadvertent nerve damage [3].

Although the prognosis of phrenic nerve palsy depends on the extent and cause of the injury, transient cases have been reported to resolve over time with supportive care and rehabilitation [4,5]. In this report, we describe a rare case of transient diaphragmatic paralysis following redo aortic valve replacement and coronary artery bypass grafting.

## Case presentation

**Figure 1:**
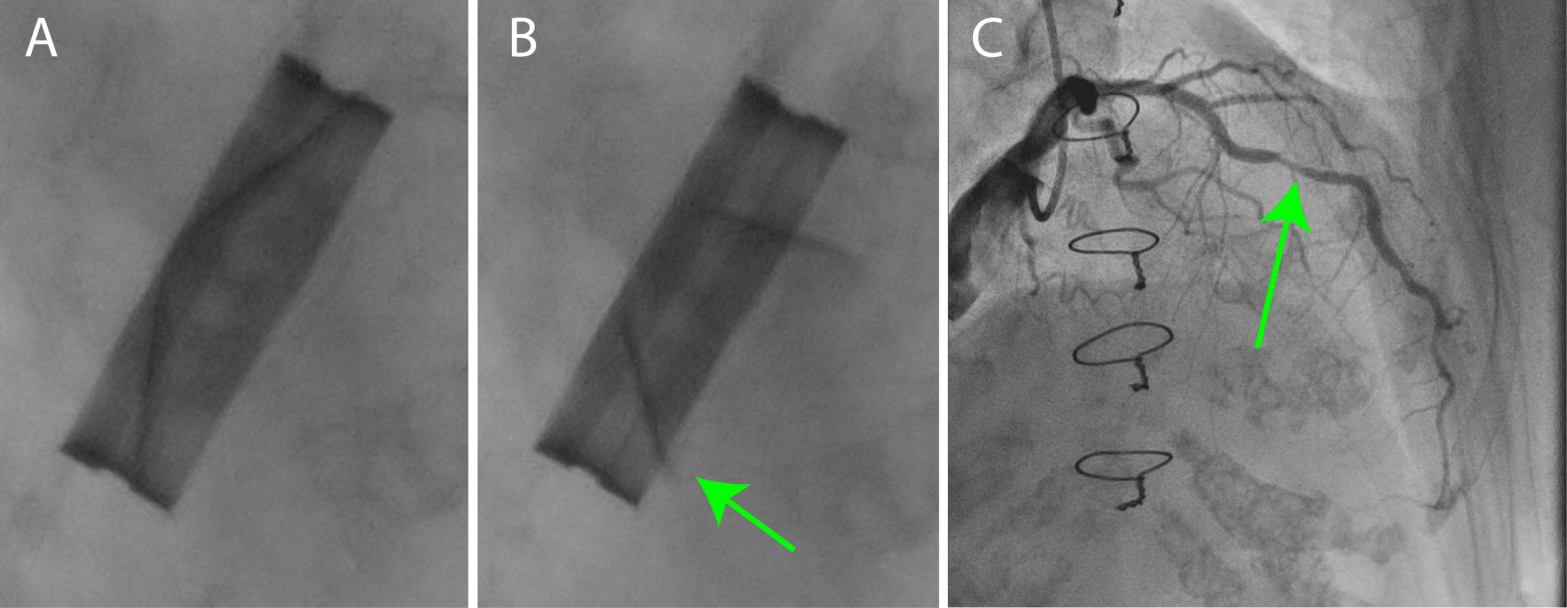
Preoperative angiography (A) Diastolic phase of aortic valve. (B) Systolic phase of aortic valve. The movement of the mechanical valve disk was faulty (green arrow). (C) Left coronary artery angiogram; 75% stenosis was detected (green arrow).

A 75-year-old woman with a history of two prior cardiac surgeries presented with progressive dyspnea on exertion. At 44 years old, she underwent aortic valve replacement (21 mm mechanical valve, St. Jude Medical^TM^) for rheumatic severe aortic stenosis. At 51 years, she underwent mitral valve replacement (29 mm mechanical valve, Carbomedics^TM^) for rheumatic mitral regurgitation. Recent transthoracic echocardiography revealed aortic stenosis with an effective orifice area of 0.79 cm² and limited opening of the mechanical valve (Figure 1). Coronary angiography demonstrated 75% stenosis in the left anterior descending artery (#6). After comprehensive discussions, the patient consented to reoperation.

**Figure 2:**
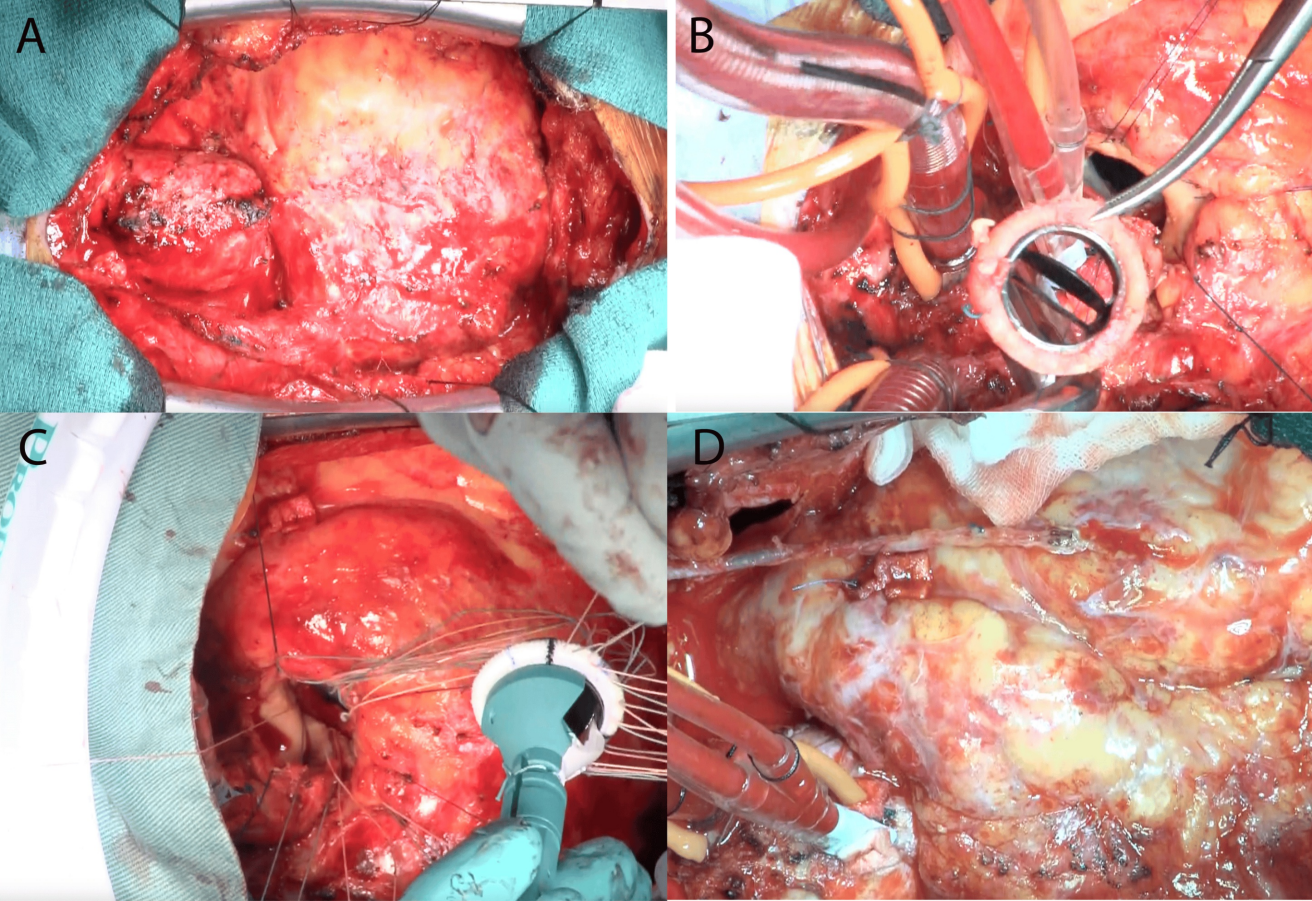
Intraoperative findings (A) Overview. External appearance. Intense adhesions between the heart and pericardium were observed. (B) Explant aortic valve. (C) New implanted aortic valve. (D) Coronary artery bypass grafting left internal thoracic artery of to left descending artery.

The operation was performed through a re-median sternotomy. Extensive pericardial adhesions, particularly around the right atrium, were meticulously lysed. The left internal thoracic artery (LITA) was harvested using an ultrasonic scalpel. Cardiopulmonary bypass was initiated following careful adhesiolysis and myocardial protection. Upon transverse aortic incision, circumferential pannus formation beneath the aortic annulus was identified and excised. The mechanical valve was explanted, and a 20 mm ATS advanced performance (AP) mechanical aortic valve (ATS Medical, Minneapolis, MN) was implanted in a supra-annular position using 14 interrupted sutures. Following aortorrhaphy, a LITA-to-left anterior descending (LAD) coronary bypass was performed (Figure 2).

The patient’s immediate postoperative course was notable for the elevated right hemidiaphragm observed on the chest X-ray. She was extubated on postoperative day 1 with stable respiratory function. However, on day 3, she developed respiratory distress, requiring reintubation and prolonged mechanical ventilation. Subsequent fluid management and supportive care enabled extubation on day 10.

On day 21, the patient aspirated sputum, necessitating reintubation. A tracheostomy was performed, and her condition stabilized. She was transferred to a rehabilitation facility on postoperative day 45. Over six months, her respiratory muscle strength improved, allowing complete weaning from ventilatory support. By her follow-up visit at six months, her right phrenic nerve paralysis had resolved, as evidenced by the normalization of diaphragmatic movement. She walked independently with a cane.

**Figure 3:**
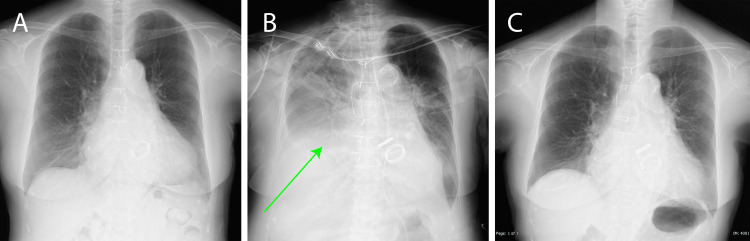
Chest X-ray imaging findings (A) Preoperative. (B) Post operative. Right diaphragm elevation (green arrow) and post-tracheostomy tube identified. (C) One year after the operation. Elevation of the right diaphragm had improved.

Two years postoperatively, the patient remains well, with no signs of respiratory or functional impairment (Figure 3). This case highlights the potential for transient phrenic nerve palsy to improve with time and appropriate management, even after complex redo cardiac surgery.

## Discussion

Phrenic nerve palsy is a rare but notable complication in cardiac surgery, particularly in redo procedures where extensive adhesiolysis and reoperative sternotomy pose higher risks of nerve injury. In this case, the patient developed transient right-sided diaphragmatic paralysis following a complex reoperation involving aortic valve replacement and coronary bypass grafting. The elevated diaphragm on imaging and subsequent respiratory distress highlighted the importance of early recognition and tailored management in such scenarios [6].

While phrenic nerve palsy can significantly impair respiratory function, this case demonstrates the potential for complete recovery over time. Transient phrenic nerve paralysis may result from ischemic, mechanical, or thermal injury during surgery [7]. Fortunately, the reversibility observed in this patient emphasizes the resilience of neural structures when spared from permanent damage. Over six months, the patient experienced substantial respiratory improvement, culminating in the resolution of diaphragmatic paralysis by her follow-up visit.

The cornerstone of the patient’s recovery was comprehensive rehabilitation. Prolonged ventilatory support, including tracheostomy, facilitated respiratory stability during acute phases. Subsequent pulmonary rehabilitation strengthened respiratory musculature and improved her functional capacity. This underscores the critical role of rehabilitation in achieving optimal outcomes for patients with transient nerve injuries post-cardiac surgery [8,9].

This case also highlights the importance of multidisciplinary care in managing complex surgical complications. Collaboration among cardiothoracic surgeons, pulmonologists, and rehabilitation specialists was pivotal in this patient’s recovery. Clinicians should maintain vigilance for phrenic nerve dysfunction in similar scenarios and prioritize individualized care strategies to maximize recovery potential.

In summary, this case illustrates the importance of recognizing and managing phrenic nerve palsy following cardiac surgery. It emphasizes the potential for nerve recovery and the vital role of rehabilitation in achieving successful long-term outcomes.

## Conclusions

Rehabilitation played a pivotal role in this case, underscoring the importance of a multidisciplinary approach to managing complications after cardiac surgery. Early and sustained rehabilitation efforts were instrumental in improving respiratory muscle strength and facilitating the patient’s recovery.

Future studies are needed to better understand the mechanisms of phrenic nerve injury during cardiac surgery and to develop strategies for prevention and accelerated recovery. By enhancing surgical techniques and postoperative care protocols, the risks of complications like phrenic nerve paralysis can be mitigated, ensuring improved long-term outcomes for patients undergoing complex cardiac procedures.
